# Potential Predictors of Pain and Stiffness Response Following Genicular Artery Embolization for Knee Osteoarthritis

**DOI:** 10.3390/jcm15051876

**Published:** 2026-02-28

**Authors:** Tarub S. Mabud, Seon-Hi Shin, Anthony Chong, Mukundan Attur, Erin Alaia, Shu Liu, Elizabeth Morris, Jonathan Samuels, William Macaulay, Bedros Taslakian

**Affiliations:** 1 Division of Vascular and Interventional Radiology, Columbia University, New York, NY 10032, USA; 2 Department of Radiology, New York University Langone Health, New York, NY 10016, USA; 3 Department of Rheumatology, New York University Langone Health, New York, NY 10016, USA; 4 Department of Orthopedic Surgery, New York University Langone Health, New York, NY 10016, USA; 5 Department of Interventional Radiology, University of Miami, Miami, FL 33136, USA; bedros.taslakian@miami.edu

**Keywords:** genicular artery embolization, osteoarthritis, knee pain

## Abstract

**Background/Objectives**: Patient-level predictors of treatment response after genicular artery embolization (GAE) for knee osteoarthritis (OA) are poorly understood. We evaluated clinical, serum, and imaging biomarkers for their ability to predict achievement of the minimally clinically important difference (MCID) for WOMAC pain and stiffness subscales following GAE. **Methods**: Data from a prospective single-arm clinical trial of 25 patients who underwent GAE for symptomatic knee OA was retrospectively analyzed. Candidate predictors included sex, age, BMI, contralateral Kellgren–Lawrence (KL) scores, and baseline values for serum IL-1Ra, serum VEGF, and total bone marrow edema scores on MRI using the MOAKS methodology. The primary outcomes were the frequency of achieving the MCID in WOMAC pain and WOMAC stiffness at 1, 3, and 12 months, modeled as an ordinal outcome (0–3). Ordinal logistic regression models were constructed. Variance inflation factors (VIFs) were assessed to detect multicollinearity, and leave-one-out cross-validation was performed to evaluate model robustness. **Results**: All candidate predictors were successfully incorporated into regression models, with no evidence of multicollinearity by VIF analysis. Lower contralateral KL scores (OR: 0.087 [0.012–0.618], *p* = 0.0146) and higher BMI (OR: 1.383 [1.001–1.910], *p* = 0.049) were significantly associated with achievement of the MCID for WOMAC pain, although significance for BMI was borderline. Lower baseline serum IL-1Ra levels (OR: 0.122 [0.018–0.816], *p* = 0.030) were significantly associated with achievement of the MCID for WOMAC stiffness. The remaining clinical, serum, and imaging biomarkers were not significantly associated with MCID achievement. **Conclusions**: In this exploratory analysis, specific baseline clinical and serum factors were associated with achievement of clinically meaningful improvements in pain and stiffness. Analysis of larger cohorts will help clarify ideal demographic-, biomarker- and imaging-based patient selection strategies that can improve prediction of treatment response and guide clinical decision-making in GAE for knee OA.

## 1. Introduction

Knee osteoarthritis (OA) is estimated to affect up to 365 million people worldwide, resulting in significant chronic disability and socioeconomic burden [1]. Per the American College of Rheumatology and Arthritis Foundation, recommended initial treatment options range from conservative management, such as weight loss, exercise, and patient-directed activity programs, to short-term symptomatic relief with intra-articular steroid injections [2]. Surgical treatment with total joint replacement is typically reserved for patients with severe knee OA. This results in a treatment gap for patients with mild-to-moderate knee OA refractory to conservative treatment options, as well as for patients with severe knee OA who are not surgical candidates [3,4]. Genicular artery embolization (GAE) is an emerging minimally invasive treatment for mild-to-moderate symptomatic knee OA refractory to conservative management, with promising initial safety and efficacy data. Multiple studies have demonstrated statistically significant improvements in patient-reported outcome measures, including the Western Ontario and McMaster Universities Osteoarthritis Index (WOMAC), the Knee Injury and Osteoarthritis Score (KOOS), the visual analog scale (VAS), and the EuroQol 5-Dimension 5-Level quality of life score (EQ-5D-5L), although sham-controlled studies have yielded mixed results [5,6,7,8,9,10,11,12,13].

OA has traditionally been considered a mechanical process driven by cartilage loss; however, there is a growing consensus that OA pathophysiology and progression are also driven by a cycle of inflammation throughout the knee joint [14,15,16]. In OA, cartilage damage leads to the release of pro-inflammatory mediators that stimulate neovascularity and nerve growth in the adjacent subchondral bone; cartilage breakdown products are also taken up by the synovium, leading to synovitis and the production of additional inflammatory mediators and catabolic enzymes that lead to further cartilage breakdown [16,17,18,19]. This inflammatory, proangiogenic, and neurotrophic cascade is thought to ultimately contribute to nociceptive peripheral pain generation [20]. GAE potentially achieves a therapeutic effect by targeting angiogenesis and decreasing the pathologic inflammation within the knee joint. GAE has been shown to reduce synovial proliferation, hypertrophy, and hyperplasia in rabbit knee models, and it has shown initial promise in treating synovial disorders such as pigmented villonodular synovitis [21,22].

Developing patient selection criteria is crucial to improving the efficacy of GAE, as certain pre-procedural/baseline criteria may predict positive or negative outcomes. Given OA’s complex, multifactorial nature, personalized treatment approaches that consider patient-specific factors, including imaging and laboratory biomarkers, can potentially improve patient outcomes and prevent unnecessary procedures for those less likely to benefit from them. Early evaluations have suggested that certain imaging criteria within the MRI Osteoarthritis Knee Score (MOAKS), such as full-thickness cartilage defects, effusion synovitis, bone marrow lesions, and osteophytes, are associated with less favorable outcomes following GAE and 6 months following the procedure [23]. In our recent single-arm prospective clinical trial evaluating longitudinal changes in serum biomarkers of inflammation after GAE, statistically significant decreases in vascular endothelial growth factor (VEGF) and interleukin-1 receptor antagonist (IL-1Ra) levels were observed 12 months following GAE [24]. The current study assesses data from this trial to preliminarily explore pre-procedural demographic, clinical, imaging, and biomarker criteria for utility as potential predictors of patient outcomes after GAE.

## 2. Materials and Methods

This retrospective exploratory analysis utilized data collected from a single-center, prospective, single-arm clinical trial evaluating GAE using 250 μm Embozene microspheres (Varian Medical Systems, Palo Alto, USA) for symptomatic knee OA (ClinicalTrials.gov identifier: NCT04379700) [24].

Between January 2021 and April 2024, consecutive patients presenting to the interventional radiology clinic for chronic knee OA pain were screened for eligibility. Inclusion criteria required ages of 30–80 years, moderate to severe radiographic knee OA (Kellgren–Lawrence [KL] grades 2–4) in the native knee, persistent knee pain for >3 months despite conservative therapy, and a baseline VAS pain score of ≥40 mm. The majority (17/25) of patients had KL 3 OA, and only 2 individuals who had KL 4 OA were not eligible for or did not desire arthroplasty.

Patients were excluded for active infection, malignancy, life expectancy < 12 months, recent knee surgery (within 3 years; arthroscopy within 6 months), prior knee arthroplasty, pregnancy, renal dysfunction, uncorrectable coagulopathy, severe contrast allergy, recent knee trauma or fracture, avascular necrosis, recent intra-articular injection (within 3 months), inflammatory arthropathies (including rheumatoid arthritis or spondyloarthropathies), lupus, or symptomatic peripheral vascular disease.

A total of 25 patients were enrolled in the original study, underwent GAE using 250 μm Embozene microspheres, and completed at least one post-procedure follow-up visit; these 25 patients constitute the analytic cohort for the present study. All patients completed 1-month follow-up, 24 completed 3-month follow-up, and 21 completed 12-month follow-up. Patient demographics (mean age: 65.2 + 7.6; 52% female; 68% white) and clinical characteristics (mean body mass index (BMI): 31.1 + 7.0; baseline KL score of target knee: 2 (24%), 3 (68%), and 4 (8%)) were published previously [24].

GAE procedures were performed by a single interventional radiologist with 8 years of experience (B.T.). Embolization targeted genicular arteries corresponding to patient-reported pain and angiographic hyperemia, with the endpoint defined as pruning of abnormal hypervascularity while preserving normal arterial flow. A detailed description of the procedural methodology was provided previously [24].

Baseline imaging included non-contrast knee MRI. MRI findings were quantified using the MOAKS by a board-certified musculoskeletal radiologist (E.A.) with 10 years of experience who was blinded to clinical data. Potential serum biomarkers of inflammation, including aggrecan, chemokine (c-c motif) ligand 2 (CCL2), C-reactive protein (CRP), interleukin-18 (IL-18), interleukin-1 receptor antagonist (IL-1Ra), interleukin-6 (IL-6), interleukin-8 (IL-8), macrophage migration inhibitory factor (MIF), beta-nerve growth factor (NGF-β), tumor necrosis factor alpha (TNF-α), and vascular endothelial growth factor (VEGF), were evaluated at baseline and at 1-, 3-, and 12-month follow-ups using validated multiplex and enzyme-linked immunosorbent assays (ELISA). Clinical outcome assessments were performed at baseline and 1-, 3-, and 12-month follow-ups after GAE and included the WOMAC, KOOS, VAS, and EQ-5D-5L scores. Institutional review board approval was obtained for the original trial under an investigational device exemption, and all participants provided written informed consent. Informed consent was waived for the current retrospective analysis.

### Statistical Analysis

The current study retrospectively analyzed pre-specified predictors of clinical improvement using data collected prospectively from the trial described above. Candidate predictors for treatment response were selected a priori by the research team after the conclusion of the original trial but before any exploratory statistical analyses were performed, and they included: sex, age, BMI, contralateral KL grade, and baseline values of the WOMAC pain score, serum IL-1Ra, serum VEGF, and the total bone marrow edema (BME) score measured by the MRI Osteoarthritis Knee Score (MOAKS) system. Comprehensive baseline demographic and clinical values were reported previously [24]; variables relevant to the current analysis are summarized in Table 1. Sex, age, and BMI were included as general demographic considerations potentially relevant for patient selection. Contralateral KL grade was included as a covariate to account for potential confounding from systemic inflammatory effects and elevated circulating biomarkers attributable to the non-treated knee. Because most patients had KL grade 3 disease in the treated knee, ipsilateral KL grade demonstrated limited variability and was therefore not considered suitable for patient stratification. Of the 10 serum biomarkers assessed in the original trial, IL-1Ra and VEGF were selected for the present analysis because they were the only potential markers of inflammation (which have previously been associated with OA progression) to demonstrate significant longitudinal changes across follow-up timepoints. Although biomarkers were prespecified as candidate predictors, restriction to these two markers was informed by prior trial findings and therefore represents a secondary, hypothesis-generating analysis that may be subject to post hoc selection bias [24]. The total BME score via the MOAKS was included as a covariate as BME has previously been identified as a potential predictor of outcomes after GAE [23].

The primary outcome was the frequency of achieving the minimal clinically important difference (MCID) in the WOMAC pain and stiffness domains at 1, 3, and 12 months post-GAE. The MCID for WOMAC pain corresponded to a >4-point reduction relative to baseline, and the MCID for WOMAC stiffness corresponded to a >2-point reduction relative to baseline [25]. Outcomes were modeled as an ordinal variable representing the cumulative number of follow-up timepoints (0–3) at which the MCID was achieved. Ordinal logistic regression models were constructed to evaluate associations between candidate predictors and this cumulative response measure. This approach reflects the extent of repeated MCID attainment across follow-up periods rather than a formally validated measure of durability and should be interpreted as exploratory. The proportional odds (parallel lines) assumption underlying the ordinal logistic regression models was assessed graphically by examining whether predictor effects appeared consistent across cumulative logits. Multicollinearity was assessed using variance inflation factors (VIFs), with VIF values > 5 indicating potential collinearity. Given the limited sample size of the original cohort, model robustness was evaluated using leave-one-out cross-validation (LOOCV), assessing the consistency and reproducible statistical significance of each predictor across the replications. A *p*-value of <0.05 was considered statistically significant for all analyses. A correction for multiple comparisons was not applied given the exploratory nature of these analyses.

## 3. Results

**Figure 1:**
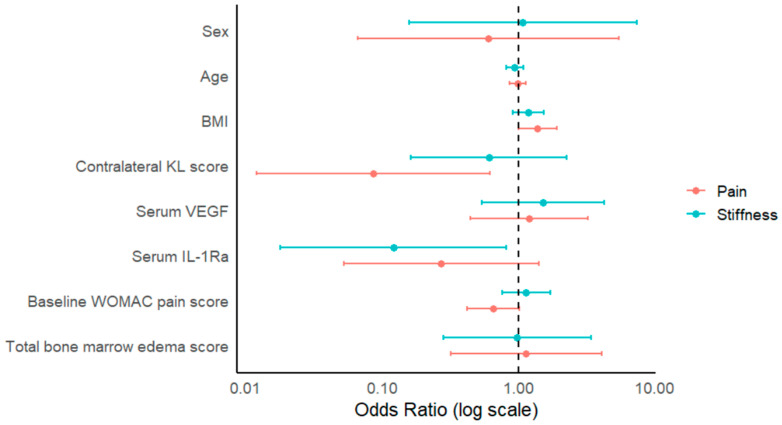
Odds ratios and 95% confidence intervals for patient characteristics associated with MCID achievement for WOMAC pain and stiffness.

Greater cumulative longitudinal MCID attainment in the WOMAC pain domain was significantly associated with contralateral KL scores (odds ratio [OR]: 0.087; 95% confidence interval [CI]: 0.012–0.618; *p* = 0.015) and BMI (OR: 1.383; 95% CI: 1.001–1.910; *p* = 0.049), although BMI was borderline in significance. Higher contralateral KL scores were associated with lower odds of cumulative MCID achievement across follow-up timepoints, whereas higher BMI was associated with higher odds of cumulative MCID achievement. For the WOMAC stiffness domain, higher baseline serum IL-1Ra levels were significantly associated with lower odds of cumulative MCID achievement across follow-up timepoints (OR: 0.122; 95% CI: 0.018–0.816; *p* = 0.030). No significant associations were observed between cumulative MCID achievement and the remaining demographic, clinical, serum, or imaging biomarkers. A summary of all predictors for both models is provided in Table 2 and graphically in Figure 1.

For regression analyses, a graphical assessment demonstrated no meaningful deviation from the proportional odds (parallel lines) assumption. All predefined candidate predictors were incorporated into the ordinal logistic regression models for WOMAC pain and WOMAC stiffness. There was no evidence of multicollinearity among predictors; VIF values ranged from 1.157 to 2.035 for both the WOMAC pain and stiffness models.

In the LOOCV analysis, among the 25 iterations, contralateral KL scores, serum IL-1Ra, and BMI were significant in 96%, 88%, and 48% of the replications, respectively. Among the nonsignificant replications for BMI, the average and median *p*-values were 0.062 and 0.058, respectively, nearing the threshold for statistical significance.

## 4. Discussion

This study was conducted as an exploratory analysis to identify potential demographic, clinical, serum, and MRI imaging biomarkers that may warrant further evaluation as predictors of treatment response following GAE, using retrospective analysis of data collected in a prior prospective clinical trial. In this cohort, regarding the WOMAC pain subscale, a lower contralateral KL score was associated with greater cumulative MCID achievement, while higher BMI demonstrated a borderline association with greater cumulative MCID achievement. Additionally, lower baseline IL-1Ra levels were associated with greater cumulative MCID achievement in the WOMAC stiffness domain.

Higher BMI demonstrated a marginal association with greater cumulative MCID attainment in WOMAC pain across follow-up periods; however, this association was not consistently reproduced in cross-validation, reaching statistical significance in only 48% of LOOCV replications. Obesity has been associated with increased systemic inflammatory biomarkers, adipokine production, synovial inflammation, and inflammation-mediated cartilage degeneration [26,27,28]. In this context, greater systemic and synovial inflammatory activity may contribute to increased periarticular hyperemia—a proposed therapeutic target of GAE. Prior studies have also evaluated BMI as a potential predictor of response after GAE, albeit with inconsistent findings [29,30]. In a prior retrospective single-center evaluation of 34 consecutive patients treated with GAE, patients with BMI < 35 had significantly higher achievement of the MCID for the WOMAC than patients with BMI ≥ 35 (41% vs. 15%; *p* = 0.063) [29]. Notably, that study dichotomized BMI at a threshold of 35 and evaluated MCID achievement as a binary outcome at fixed follow-up intervals over one and two years. In contrast, Callese et al. evaluated 236 patients and defined clinical success as a 50% reduction in WOMAC score, analyzing BMI as a continuous variable and reporting an association between higher BMI and an increased likelihood of response [30]. Differences in outcome definitions (MCID vs. 50% WOMAC reduction), modeling strategy (binary vs. continuous predictors), sample size, and follow-up duration may partially explain the conflicting findings across studies. Additionally, variations in disease severity and patient selection criteria may further influence the observed relationship between BMI and GAE outcomes. Given these methodological differences and the marginal, inconsistently replicated association observed in the present analysis, BMI should be considered a hypothesis-generating variable that warrants validation in larger cohorts with standardized outcome definitions.

A lower contralateral KL score indicates the presence of less advanced knee OA in the untreated knee, which was associated with greater cumulative MCID achievement in WOMAC pain scores after GAE. Although this finding should be interpreted cautiously given wide confidence intervals, it raises the possibility that less severe contralateral disease may reduce compensatory biomechanical stress on the treated knee. Conversely, patients with advanced contralateral OA may experience altered gait mechanics and increased load transfer, potentially limiting symptomatic improvement after GAE [31].

Among serum biomarkers, lower baseline levels of IL-1Ra were associated with greater cumulative MCID attainment in WOMAC stiffness. IL-1Ra is an endogenous antagonist that counteracts the pro-inflammatory effects of IL-1, a cytokine implicated in cartilage degradation and synovial inflammation [32]. One speculative explanation is that lower circulating IL-1Ra levels may reflect a distinct inflammatory phenotype in which synovial hyperemia and cytokine-driven processes are more prominent and therefore more amenable to embolization-mediated modulation. However, serum IL-1Ra represents only an indirect surrogate of intra-articular cytokine activity, and the relationship between systemic cytokine levels and local joint inflammation is incompletely understood. Importantly, this observational association does not imply a causal role for IL-1Ra in mediating treatment response. The roles of IL-1 and IL-1Ra in osteoarthritis pathophysiology remain controversial, with conflicting findings reported in the literature regarding the clinical impact of targeted cytokine modulation [33].

Our study did not demonstrate any pre-procedural MRI findings associated with positive or negative clinical outcomes following GAE. MOAKS has previously been studied in the context of GAE, demonstrating that certain MRI findings, including full-thickness cartilage defects, effusion synovitis, bone marrow lesions, and osteophytes, were associated with less favorable GAE outcomes at 6 months [23]. This discrepancy may be related to several factors, including sample size constraints and potential differences in MRI acquisition parameters. Additionally, while MOAKS scoring in the present study was performed by an experienced board-certified musculoskeletal radiologist who was blinded to clinical data, formal interobserver reproducibility assessment was not performed. Given that semi-quantitative scoring systems such as MOAKS are inherently reader-dependent, interobserver variability may influence the stability of imaging biomarker associations, particularly in smaller exploratory cohorts. Therefore, the predictive value of MRI findings warrants further evaluation in larger cohorts with standardized imaging approaches and longer follow-up durations to clarify their role in procedural selection and outcome stratification.

This study has several limitations. The number of covariates chosen for multivariate ordinal regression and the resulting analyses are limited by sample size constraints. However, this study aims to preliminarily identify potential predictive patient characteristics and/or biomarkers for further exploration in subsequent larger studies featuring greater statistical power. To mitigate sample size constraints, VIF and LOOCV analyses were performed to evaluate multicollinearity between predictors and model robustness; no predictor variables exceeded the traditional VIF threshold of five, supporting the independence of the included covariates. While VIF analysis did not demonstrate multicollinearity, this does not eliminate the risk of overfitting or coefficient instability associated with the small sample size. The limited events-per-variable ratio remains as an important constraint, and the results should be interpreted cautiously. For predictors that were also not consistently significant across iterations in LOOCV analyses, the average and median *p*-values in non-significant replications remained near conventional significance thresholds, supporting their relative contributions to model performance. These results suggest model stability for the most influential predictors. As the data was initially collected as part of a prospective clinical trial, findings are not fully generalizable to real-world patient populations due to selection bias related to initial inclusion criteria and additionally due to single-center design and single-operator performance. The lack of a control arm in the original trial precludes differentiation of the true treatment effect from placebo and contextual effects. Moreover, while utilization of MCID achievement at multiple timepoints captures cumulative clinical benefit across follow-up durations, it does not fully characterize temporal patterns of response, which may be better addressed in future longitudinal analyses; longitudinal mixed-effects models or time-to-event analyses might provide complementary insights for future studies with larger cohorts. As noted previously, interpretation of serum biomarkers is constrained by an incomplete understanding of their relationship to synovial fluid biomarkers.

This study identifies several exploratory associations with outcomes after GAE based on data derived from a single-arm prospective clinical trial. While these findings are preliminary and hypothesis-generating, they suggest a potential framework for future risk stratification approaches. For example, if validated, demographic factors such as BMI, contralateral disease burden, or inflammatory biomarker profiles could eventually contribute to multivariable prediction models designed to estimate the likelihood of clinically meaningful improvement following GAE. In real-world practice, such models could support shared decision-making by identifying patients more likely to benefit from embolization versus alternative interventions. Future studies are needed to further investigate these potentially predictive patient characteristics or biomarkers in larger cohorts to optimize patient selection and care of patients with knee OA.

## Figures and Tables

**Table 1:** Patient demographic and clinical characteristics included in the ordinal logistic regression model.

Baseline Characteristic	Value
Sex, n (%)	
Female	13 (52)
Male	12 (48)
Age, mean + SD (y)	65.2 + 7.6
BMI, mean + SD	31.1 + 7.0
Contralateral Kellgren–Lawrence score, n (%)	
0	1 (4)
1	4 (16)
2	8 (32)
3	10 (40)
4	2 (8)
Serum VEGF, mean + SD (pg/mL)	302.7 + 326.7
Serum IL-1Ra, mean + SD (pg/mL)	833.4 + 425.8
WOMAC pain score	8.6 + 2.3
Total bone marrow edema score	5.4 + 3.6

**Table 2:** Associations between patient characteristics and MCID achievement for WOMAC pain and stiffness.

Baseline Predictor Variables		Pain			Stiffness	
	OR ^a^	95% C.I.	*p* ^b^	OR	95% C.I.	*p*
Sex Male Female (reference)	0.604	[0.067–5.447]	0.653	1.084	[0.159–7.378]	0.934
Age	0.993	[0.861–1.145]	0.921	0.944	[0.815–1.093]	0.439
BMI	1.383	[1.001–1.910]	0.049	1.182	[0.910–1.536]	0.210
Contralateral Kellgren–Lawrence score	0.087	[0.012–0.618]	0.015	0.608	[0.163–2.264]	0.458
Serum VEGF	1.202	[0.447–3.232]	0.715	1.519	[0.543–4.253]	0.426
Serum IL-1Ra	0.273	[0.053–1.417]	0.122	0.122	[0.018–0.816]	0.030
WOMAC pain score	0.658	[0.424–1.022]	0.062	1.143	[0.759–1.722]	0.522
Total bone marrow edema score	1.138	[0.318–4.071]	0.842	0.984	[0.281–3.446]	0.980

^a^ Odds ratio; ^b^ *p*-value. Note. Results of ordinal logistic regression for two categorical outcome variables are presented.

## Data Availability

The datasets presented in this article are not readily available because the data are part of an ongoing study. Requests to access the datasets should be directed to B.T.

## Abbreviations

The following abbreviations are used in this manuscript:

GAE	Genicular artery embolization
OA	Osteoarthritis
MCID	Minimal clinically important difference
BMI	Body mass index
VEGF	Vascular endothelial growth factor
IL-1Ra	Interleukin-1 receptor antagonist
KL	Kellgren-Lawrence
WOMAC	Western Ontario and McMaster Universities Osteoarthritis Index
OR	Odds ratio
KOOS	Knee Injury and Osteoarthritis Score
VAS	Visual analog scale
MOAKS	Magnetic resonance imaging Osteoarthritis Knee Score
EQ-5D-5L	EuroQol 5-Dimension 5-Level quality of life score
ELISA	Enzyme-linked immunosorbent assay
LOOCV	Leave-one-out cross-validation
VIF	Variance inflation factor
CI	Confidence interval
CCL2	Chemokine (c-c motif) ligand 2
CRP	C-reactive protein
MIF	Macrophage migration inhibitory factor
NGF-β	Nerve growth factor beta
TNF-α	Tumor necrosis factor alpha

## References

[1] Leifer V.P., Katz J.N., Losina E. (2022). The burden of OA-health services and economics. Osteoarthr. Cartil..

[2] Buelt A., Narducci D.M. (2021). Osteoarthritis Management: Updated Guidelines from the American College of Rheumatology and Arthritis Foundation. Am. Fam. Physician.

[3] London N.J., Miller L.E., Block J.E. (2011). Clinical and economic consequences of the treatment gap in knee osteoarthritis management. Med. Hypotheses.

[4] Allen K.D., Golightly Y.M., White D.K. (2017). Gaps in appropriate use of treatment strategies in osteoarthritis. Best Pract. Res. Clin. Rheumatol..

[5] Okuno Y., Korchi A.M., Shinjo T., Kato S., Kaneko T. (2017). Midterm Clinical Outcomes and MR Imaging Changes after Transcatheter Arterial Embolization as a Treatment for Mild to Moderate Radiographic Knee Osteoarthritis Resistant to Conservative Treatment. J. Vasc. Interv. Radiol..

[6] Taslakian B., Swilling D., Attur M., Alaia E.F., Kijowski R., Samuels J., Macaulay W., Ramos D., Liu S., Morris E.M. (2023). Genicular Artery Embolization for Treatment of Knee Osteoarthritis: Interim Analysis of a Prospective Pilot Trial Including Effect on Serum Osteoarthritis-Associated Biomarkers. J. Vasc. Interv. Radiol..

[7] Bagla S., Piechowiak R., Hartman T., Orlando J., Del Gaizo D., Isaacson A. (2020). Genicular Artery Embolization for the Treatment of Knee Pain Secondary to Osteoarthritis. J. Vasc. Interv. Radiol..

[8] Padia S.A., Genshaft S., Blumstein G., Plotnik A., Kim G.H.J., Gilbert S.J., Lauko K., Stavrakis A.I. (2021). Genicular Artery Embolization for the Treatment of Symptomatic Knee Osteoarthritis. JBJS Open Access.

[9] Little M.W., Gibson M., Briggs J., Speirs A., Yoong P., Ariyanayagam T., Davies N., Tayton E., Tavares S., MacGill S. (2021). Genicular artEry embolizatioN in patiEnts with oSteoarthrItiS of the Knee (GENESIS) Using Permanent Microspheres: Interim Analysis. Cardiovasc. Interv. Radiol..

[10] Taslakian B., Miller L.E., Mabud T.S., Macaulay W., Samuels J., Attur M., Alaia E.F., Kijowski R., Hickey R., Sista A.K. (2023). Genicular artery embolization for treatment of knee osteoarthritis pain: Systematic review and meta-analysis. Osteoarthr. Cartil. Open.

[11] Bagla S., Piechowiak R., Sajan A., Orlando J., Hartman T., Isaacson A. (2022). Multicenter Randomized Sham Controlled Study of Genicular Artery Embolization for Knee Pain Secondary to Osteoarthritis. J. Vasc. Interv. Radiol..

[12] Landers S., Hely R., Hely A., Harrison B., Page R.S., Maister N., Gwini S.M., Gill S.D. (2023). Genicular artery embolization for early-stage knee osteoarthritis: Results from a triple-blind single-centre randomized controlled trial. Bone Jt. Open.

[13] van Zadelhoff T.A., Bos P.K., Moelker A., Bierma-Zeinstra S.M.A., van der Heijden R.A., Oei E.H.G. (2024). Genicular artery embolisation versus sham embolisation for symptomatic osteoarthritis of the knee: A randomised controlled trial. BMJ Open.

[14] Loeser R.F., Goldring S.R., Scanzello C.R., Goldring M.B. (2012). Osteoarthritis: A disease of the joint as an organ. Arthritis Rheum..

[15] Berenbaum F. (2013). Osteoarthritis as an inflammatory disease (osteoarthritis is not osteoarthrosis!). Osteoarthr. Cartil..

[16] Bonnet C.S., Walsh D.A. (2005). Osteoarthritis, angiogenesis and inflammation. Rheumatology.

[17] Walsh D.A., McWilliams D.F., Turley M.J., Dixon M.R., Fransès R.E., Mapp P.I., Wilson D. (2010). Angiogenesis and nerve growth factor at the osteochondral junction in rheumatoid arthritis and osteoarthritis. Rheumatology.

[18] Hu Y., Chen X., Wang S., Jing Y., Su J. (2021). Subchondral bone microenvironment in osteoarthritis and pain. Bone Res..

[19] Thomson A., Hilkens C.M.U. (2021). Synovial Macrophages in Osteoarthritis: The Key to Understanding Pathogenesis?. Front. Immunol..

[20] Mapp P.I., Walsh D.A. (2012). Mechanisms and targets of angiogenesis and nerve growth in osteoarthritis. Nat. Rev. Rheumatol..

[21] Ro D.H., Jang M.-J., Koh J., Choi W.S., Kim H.-C., Han H.-S., Choi J.W. (2023). Mechanism of action of genicular artery embolization in a rabbit model of knee osteoarthritis. Eur. Radiol..

[22] Cappucci M., Totti R., Bocchino G., Comodo R.M., Capece G., Rinaldi P.M., De Santis V. (2025). Genicular Artery Embolization with Imipenem/Cilastatin for Pigmented Villonodular Synovitis of the Knee: A Case Report. Surgeries.

[23] van Zadelhoff T.A., Okuno Y., Bos P.K., Bierma-Zeinstra S.M.A., Krestin G.P., Moelker A., Oei E.H.G. (2021). Association between Baseline Osteoarthritic Features on MR Imaging and Clinical Outcome after Genicular Artery Embolization for Knee Osteoarthritis. J. Vasc. Interv. Radiol..

[24] Taslakian B., Mabud T., Attur M., Alaia E.F., Samuels J., Macaulay W., Ramos D., Salami C., Liu S., Morris E.M. (2025). A Prospective Single-Arm Trial of Genicular Artery Embolization for Symptomatic Knee Osteoarthritis: Clinical and Biomarker Outcomes. J. Vasc. Interv. Radiol..

[25] Kim M.S., Koh I.J., Choi K.Y., Sung Y.G., Park D.C., Lee H.J., In Y. (2021). The Minimal Clinically Important Difference (MCID) for the WOMAC and Factors Related to Achievement of the MCID After Medial Opening Wedge High Tibial Osteotomy for Knee Osteoarthritis. Am. J. Sports Med..

[26] Monteiro R., Azevedo I. (2010). Chronic Inflammation in Obesity and the Metabolic Syndrome. Mediat. Inflamm..

[27] Sanchez-Lopez E., Coras R., Torres A., Lane N.E., Guma M. (2022). Synovial inflammation in osteoarthritis progression. Nat. Rev. Rheumatol..

[28] Pottie P., Presle N., Terlain B., Netter P., Mainard D., Berenbaum F. (2006). Obesity and osteoarthritis: More complex than predicted!. Ann. Rheum. Dis..

[29] Badar W., Al-Qawasmi F., Alkhani L., Varadhan A., Sajan A., Yu Q., Anitescu M., Ross B., Wallace S., Patel M. (2025). One- and Two-Year Outcomes of Genicular Artery Embolization for Symptomatic Knee Osteoarthritis: A Single-Center, Retrospective Study Using 200-µm Polyethylene Glycol Microspheres. Cardiovasc. Interv. Radiol..

[30] Callese T., Cusumano L.R., Sparks H., Masterson K., Genshaft S., Stewart J.K., Padia S.A. (2025). Early intervention in knee osteoarthritis with genicular artery embolization is associated with improved clinical outcomes. Eur. Radiol..

[31] Creaby M.W., Bennell K.L., Hunt M.A. (2012). Gait Differs Between Unilateral and Bilateral Knee Osteoarthritis. Arch. Phys. Med. Rehabil..

[32] Arend W.P. (2002). The balance between IL-1 and IL-1Ra in disease. Cytokine Growth Factor Rev..

[33] Vincent T.L. (2019). IL-1 in osteoarthritis: Time for a critical review of the literature. F1000Research.

